# Effect of electron-withdrawing fluorine and cyano substituents on photovoltaic properties of two-dimensional quinoxaline-based polymers

**DOI:** 10.1038/s41598-021-03763-1

**Published:** 2021-12-21

**Authors:** Seok Woo Lee, MD. Waseem Hussain, Sanchari Shome, Su Ryong Ha, Jae Taek Oh, Dong Ryeol Whang, Yunseul Kim, Dong-Yu Kim, Hyosung Choi, Dong Wook Chang

**Affiliations:** 1 Department of Industrial Chemistry, Pukyong National University, 48513 Busan, Republic of Korea; 2 Department of Chemistry, Research Institute for Natural Science and Institute of Nano Science and Technology, Hanyang University, 04730 Seoul, Republic of Korea; 3 Department of Advanced Materials, Hannam University, Daejeon, 34054 Republic of Korea; 4 School of Materials Science and Engineering (SMSE), Research Institute of Solar and Sustainable Energies (RISE), Gwangju Institute of Science and Technology (GIST), Gwangju, 61005 Republic of Korea

**Keywords:** Electronic devices, Solar cells

## Abstract

In this study, strong electron-withdrawing fluorine (F) and cyano (CN) substituents are selectively incorporated into the quinoxaline unit of two-dimensional (2D) D–A-type polymers to investigate their effects on the photovoltaic properties of the polymers. To construct the 2D polymeric structure, electron-donating benzodithiophene and methoxy-substituted triphenylamine are directly linked to the horizontal and vertical directions of the quinoxaline acceptor, respectively. After analyzing the structural, optical, and electrochemical properties of the resultant F- and CN-substituted polymers, labeled as PBCl-MTQF and PBCl-MTQCN, respectively, inverted-type polymer solar cells with a non-fullerene Y6 acceptor are fabricated to investigate the photovoltaic performances of the polymers. It is discovered that the maximum power conversion efficiency of PBCl-MTQF is 7.48%, whereas that of PBCl-MTQCN is limited to 3.52%. This significantly reduced PCE of the device based on PBCl-MTQCN is ascribed to the formation of irregular, large aggregates in the active layer, which can readily aggravate the charge recombination and charge transport kinetics of the device. Therefore, the photovoltaic performance of 2D quinoxaline-based D–A-type polymers is significantly affected by the type of electron-withdrawing substituent.

## Introduction

The unique advantages of polymer solar cells (PSCs) such as semi-transparent, flexible, light weight, and feasible for scalable roll-to-roll processes render them attractive as a promising green and renewable energy resource^1–3^. In a typical PSC, an *n*-type acceptor and *p*-type polymeric donor form a bulk heterojunction (BHJ) network with large interfacial areas to facilitate charge separation and charge transport^4^. Although fullerene derivatives are the most widely used *n*-type acceptors in recent decades, recent breakthroughs pertaining to non-fullerene acceptors (NFAs) have rapidly changed the paradigm. Compared with fullerene-type counterparts, NFAs possess more effective features such as a tunable electronic structure, broad light absorption, and good morphology for improving device performance^5–7^. Hence, the power conversion efficiency (PCE) of state-of-the-art NFA-based PSCs has exceeded 18%^8^.

In addition to the significant progress in *n*-type acceptors, the development of *p*-type polymeric donors with appropriate energy levels, good carrier mobility, and favorable morphologies for realizing high-performance PSCs has been emphasized^9^. These polymers typically comprise alternating electron-donating (D) and electron-accepting (A) units along the polymer chain, in which a useful intramolecular charge transfer (ICT) state is readily generated. Among the various promising building blocks for D–A type polymers, the quinoxaline moiety has emerged as a promising “A” component because of its unique merits, such as simple synthetic routes and facile structural variations^10^. Additionally, its mild electron-accepting ability facilitates the preparation of medium-bandgap polymers. The combination of medium-bandgap polymeric donors and narrow-bandgap NFAs allows BHJ photoactive layers to exhibit beneficial complementary light absorption profiles^11^. Moreover, it was recently discovered that the incorporation of strong electron-withdrawing substituents into D and/or A units can efficiently improve the photovoltaic performance of the corresponding D–A-type polymers^12–14^. In this regard, several quinoxaline-based D–A-type polymers possessing electron-withdrawing substituents have been investigated to achieve high-performance nonfullerene PSCs^15–18^. However, more systematic studies associated with this interesting topic are still required because the effects of the electron-withdrawing substituents on the photovoltaic properties of the polymers are highly susceptible to the chemical composition and configuration of the polymer structure.

In this study, two-dimensional (2D) D-A type polymers with strong electron-withdrawing fluorine (F) atoms or cyano (CN) units on electron-accepting quinoxaline acceptors were designed and synthesized. As the representative electron-withdrawing substituent, fluorine atom was firstly considered. It can efficiently reduce the energy levels of the highest occupied molecular orbital (HOMO) and lowest unoccupied molecular orbital (LUMO) of conjugated polymers with minimized steric hindrance^12^. Meanwhile, the stronger electron-withdrawing CN substituent was selected as the counterpart of F atom, because CN substituent can provide the differentiated features on the polymers such as the higher dipole moments and reduced bandgaps by mainly stabilizing LUMO energy levels^19–21^. Therefore, the studies on the effect of F and CN group on the photovoltaic properties of 2D D-A type polymers will be of great importance. To develop target polymers, thiophene-substituted benzodithiophene (BDT) units were selected as the primary electron-donating building blocks because of their advantages such as good electron-donating capability, high planarity, and less steric hindrance^22^. In addition, an electron-withdrawing chlorine (Cl) atom was introduced at the 4-position of the thiophene substituents on the BDT donor. This is because the insertion of Cl into BDT donor can induce positive effects on the photovoltaic performances of the resultant D-A type polymers^23,24^. Subsequently, quinoxaline with two methoxy-substituted triphenylamine (methoxy-TPA) moieties on its 2,3-positions was adopted as the electron-accepting component. The two existing methoxy-TPA moieties can serve as additional electron donors in the vertical direction of the quinoxaline acceptor. Moreover, the strong electron-withdrawing F or CN moiety was selectively incorporated at the 5-position of the quinoxaline acceptor to analyze their effects on the photovoltaic properties of the polymers. Finally, the polymerization of the BDT donor with F- and CN-substituted quinoxaline acceptors, denoted as PBCl-MTQF and PBCl-MTQCN, respectively, can yield the desired polymers. By employing the well-known fused-ring-type small molecule Y6 as an *n*-type NFA^25^, inverted-type devices with the structure of indium tin oxide (ITO)/SnO/polymer:Y6/MoO_3_/Ag were fabricated. The power conversion efficiency (PCE) of the fluorinated PBCl-MTQF was 7.48%, whereas that of the CN-substituted PBCl-MTQCN was limited to 3.52%. These results clearly demonstrate the significant effect of the strong electron-withdrawing F and CN substituents on the photovoltaic properties of the corresponding two-dimensional (2D) quinoxaline-based polymers.

## Results and discussion

### Synthesis and characterization of polymers

**Figure 1:**
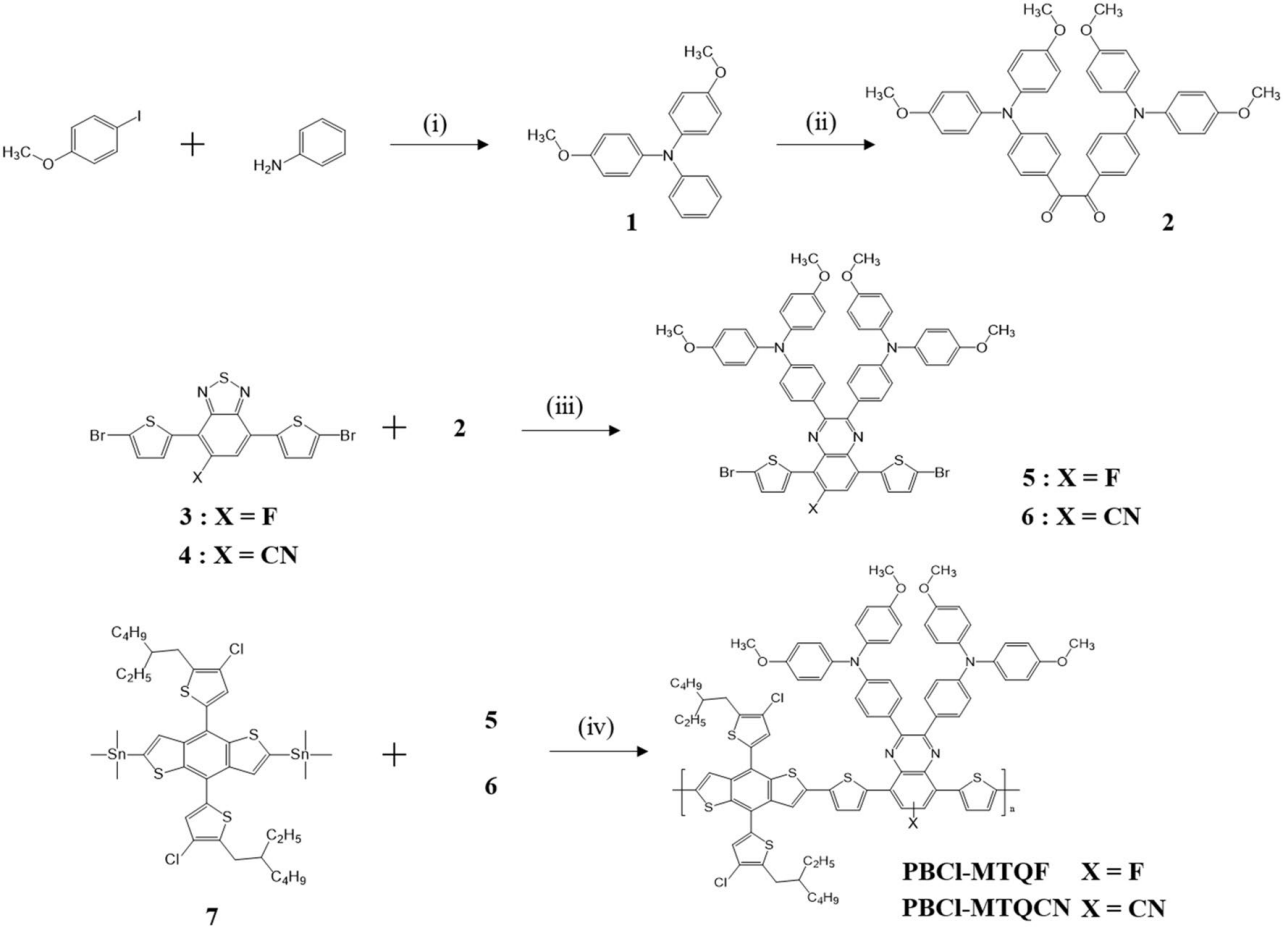
Synthesis of monomers and polymers: (i) CuI, KOH, 1,10-phenanthroline, toluene, 90 °C, 24 h; (ii) AlCl_3_, oxalyl chloride, dichloromethane, 0 °C, 1 h, and then at room temperature for 12 h; (iii) zinc, acetic acid, 80 °C, 4 h, and then 2, acetic acid, overnight; (iv) Pd_2_(dba)_3_, chlorobenzene/dimethylformamide, 110 °C, 48 h.

The preparation routes for the monomers and polymers are shown in Fig. 1, and their synthetic details and analytical results are described in the experimental section of the Electronic Supporting Information (ESI). First, the reaction of 1-iodo-4-methoxybenzene with aniline-afforded *N*,*N*-bis(4-methoxyphenyl)benzenamine (1) was performed. Second, *α*-diketone 1,2-bis(4-(bis(4-methoxyphenyl)amino)phenyl)ethane-1,2-dione (2) was prepared via the Friedel–Crafts reaction of 1 in the presence of oxalyl chloride^26^. Third, two reactions, i.e., zinc-mediated reduction and condensation, were performed on 4,7-dibromo[*c*][1,2,5]thiadiazole derivatives (3 and 4) to synthesize dibrominated quinoxaline monomers with strong electron-withdrawing F (5) and CN substituents (6). Finally, the polymerization of a chlorinated BDT donor (7) with 5 and 6 under the Stille coupling condition yielded the target D–A-type quinoxaline-based polymers, i.e., PBCl-MTQF and PBCl-MTQCN, respectively. In this synthetic strategy, a unique 2D polymeric architecture, in which the electron-donating BDT and methoxy-TPA groups are located in the horizontal and vertical directions of the electron-accepting quinoxaline unit, respectively, was achieved. The formation of the 2D structure not only strengthened the structural uniqueness of the target polymers, but also induced broad light absorption and reduced the bandgap through the facile electron transfer process in both directions. The molecular weights of the polymers were analyzed via gel permeation chromatography using *o*-dichlorobenzene as the eluent. The number-average molecular weight and polydispersity index of PBCl-MTQF and PBCl-MTQCN were 20.82 kDa and 2.48, and 26.23 kDa and 2.24, respectively. These polymers exhibited good solubility in various organic solvents such as chloroform, toluene, and chlorobenzene, owing to the existence of 2-ethylhexyl and methoxy side chains on BDT and quinoxaline units, respectively.

### Optical and electrochemical properties

**Figure 2:**
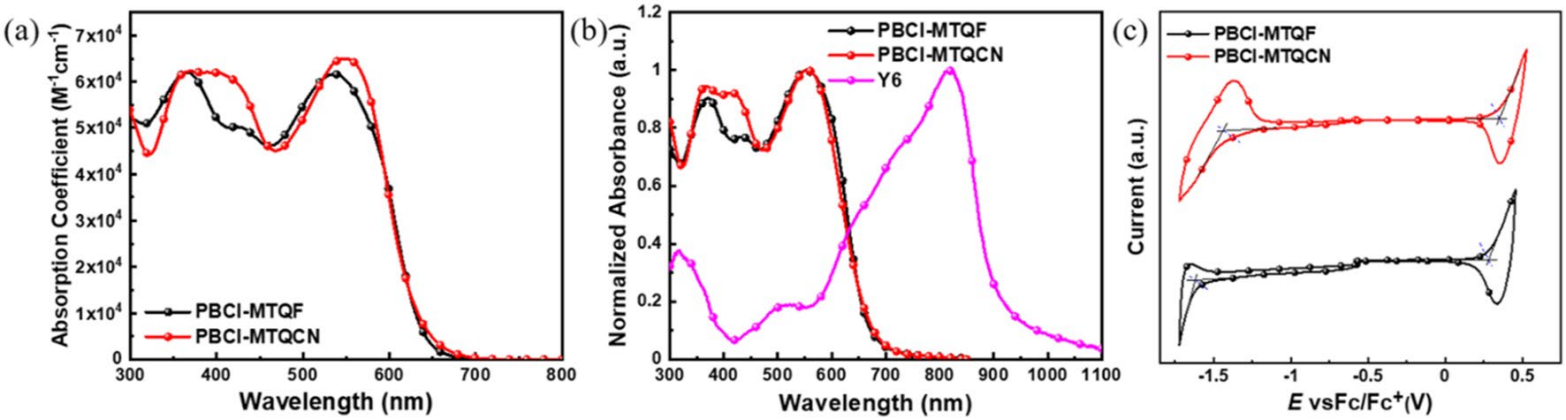
(**a**) UV–Vis spectra of polymers in chloroform solution and (**b**) polymers and Y6 in film on glass substrate. (**c**) CV curves of polymers.

The optical properties of the polymers were analyzed using ultraviolet–visible (UV–Vis) spectroscopy. As shown in Fig. 2a,b, all polymers exhibited two similar absorption peaks in both the chloroform solution and film on the glass substrate. The peak in the shorter wavelength region of 300–450 nm was associated with the π–π* transitions of the conjugated backbones, whereas that at longer wavelength regions of 450–650 nm originated from the formation of an ICT state between the donor and acceptor units in the polymer chains. The molar extinction coefficients (*ε*) in the ICT region of PBCl-MTQF and PBCl-MTQCN in chloroform solution were 6.16 × 10^4^ and 6.50 × 10^4^ M^−1^ cm^−1^, respectively (Fig. 2a). The maximum absorption peak of PBCl-MTQCN was red shifted by approximately 10 nm compared with that of PBCl-MTQF. The higher *ε* value of PBCl-MTQCN at longer wavelengths compared with that of PBCl-MTQF can be attributed to the stronger ICT formation caused by the availability of more electron-withdrawing CN units^27^. The electronic effects of the substituent can be correlated with the Hammett constant, and the values for F and CN at meta-positions were determined to be 0.34 and 0.56, respectively^28^. The two polymers exhibited good complementary light absorption spectra with an *n*-type Y6 acceptor in the film state (Fig. 2b), which is beneficial for improving the photovoltaic properties of the devices. In addition, the optical bandgaps of PBCl-MTQF and PBCl-MTQCN calculated from the absorption edge in film state were 1.81 and 1.80 eV, respectively.

The electrochemical properties of the polymers were analyzed using cyclic voltammetry (CV) measurements with a ferrocene (Fc)/ferrocenium (Fc^+^) external standard. The HOMO and LUMO energy levels were determined from the onset oxidation and reduction potentials, respectively, in the CV curves (Fig. 2c). The calculated HOMO/LUMO energy levels of PBCl-MTQF and PBCl-MTQCN were − 5.06/− 3.27 and − 5.14/− 3.38 eV, respectively. The higher electron-withdrawing capability of the CN unit compared with that of the F atom in the quinoxaline acceptor can induce a significant reduction in both the HOMO and LUMO energy levels of PBCl-MTQCN compared with those of PBCl-MTQF. In addition, the electrochemical bandgaps of PBCl-MTQF and PBCl-MTQCN estimated from the difference between their HOMO and LUMO energy levels were 1.79 and 1.76 eV, respectively. The electrochemical bandgap of the polymers exhibited the same trend as the optical bandgaps. The optical and electrochemical properties of the polymers are listed in Table 1. Based on the results, it was discovered that the replacement of the F atom with the CN moiety in the quinoxaline acceptor significantly affected the optical and electrochemical properties of the corresponding D–A-type polymers.

**Table 1:** Optical and electrochemical properties of the polymers.

Polymers	*ε* (M^−1^ cm^−1^)	${E}_{gap}^{opt}$ ^a^ (eV)	${\lambda }_{max}^{solution}$(nm)^b^	HOMO (eV)^c^	LUMO (eV)^d^	${E}_{gap}^{elec}$(eV)^e^
PBCl-MTQF	6.16 $\times$ 10^4^	1.81	366, 536	− 5.06	− 3.27	1.79
PBCl-MTQCN	6.50 $\times$ 10^4^	1.80	370, 548	− 5.14	− 3.38	1.76

^a^Estimated from the absorption edge in the film state.

^b^Maximum absorption wavelengths of polymers in chloroform solution.

^c^Estimated from the oxidation onset potential.

^d^Estimated from the reduction onset potential.

^e^Calculated from the oxidation and reduction onset potentials in the CV curves.

### Theoretical calculation

**Figure 3:**
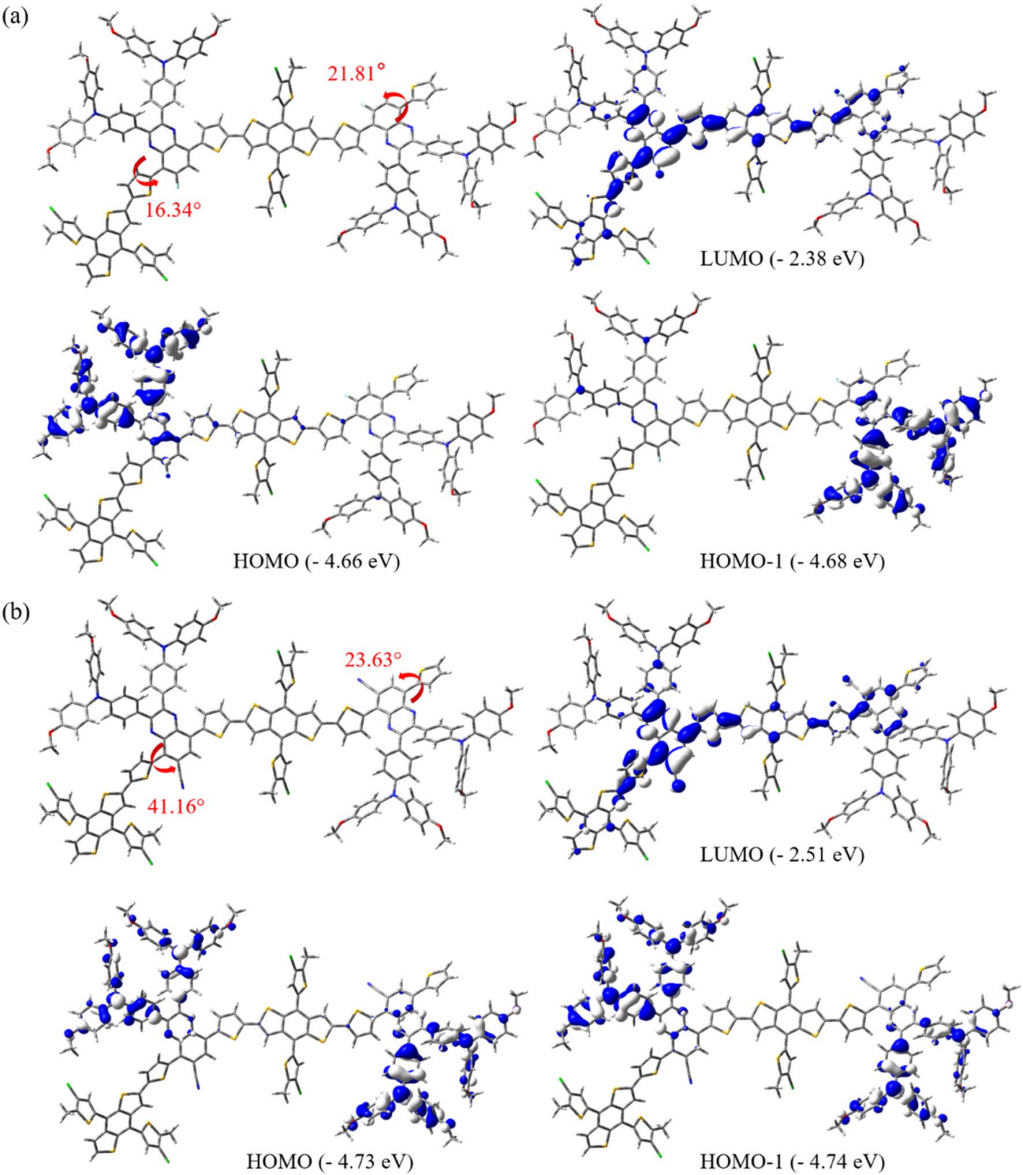
Optimized geometries and frontier molecular orbitals of two-repeating unit calculated energy levels at B3LYP/6-31G** level for (**a**) PBCl-MTQF and (**b**) PBCl-MTQCN.

Density functional simulations using the Gaussian 09 program at the B3LYP/6-31G** level were performed to estimate the optimized geometries and frontier molecular orbitals of the polymers^29^. To reduce complicated computational calculations, 2-ethylhexyl chains on BDT donors and long polymer backbones were simplified to the shortest methyl group and small dimer unit, respectively. In the optimized geometries, the dihedral angles between the thiophene unit and quinoxaline acceptor in the polymer backbone changed significantly from 16.34° in PBCl-MTQF to 41.16° in PBCl-MTQCN. The increased steric hindrance induced by the substitution of the bulkier CN moiety instead of the F atom can readily generate more twisted conformation in PBCl-MTQCN. Contrastingly, the dihedral angles between the quinoxaline acceptor and the thiophene unit on the other side are virtually identical due to their constitutional similarity; their effect on the electronic structures can be minimized (Fig. 3). From the viewpoint of frontier molecular orbitals, similar characteristic features were observed in the two polymers. The HOMO wave functions of all the polymers were localized on the methoxy-TPA units, whereas their LUMO wave functions were delocalized along the polymer backbones. However, the HOMO and LUMO energy levels of the polymers were altered significantly by the type of electron-withdrawing substituent on the quinoxaline acceptor. The simulated HOMO/LUMO energy levels of PBCl-MTQF and PBCl-MTQCN were − 4.66/− 2.38 eV and − 4.73/− 2.51 eV, respectively. In addition, HOMO and HOMO-1 are doubly degenerated, with energy differences of 20 meV and 10 meV for PBCl-MTQF and PBCl-MTQCN, respectively. The HOMO/HOMO-1 wave functions of all the polymers were localized on the methoxy-TPA units, whereas their LUMO wave functions were delocalized along the polymer backbones. So, both the HOMO and LUMO energy levels of PBCl-MTQCN were more stable than those of PBCl-MTQF. As experimentally observed based on the HOMO/LUMO energy levels of the polymers using CV measurements, the theoretical HOMO/LUMO energy levels of the polymers reduced owing to the substitution of F atom with CN moiety.

## Methods

### Fabrication and analysis of the devices

The ITO glass substrate was cleaned via sequential sonication with deionized water, acetone, and 2-propanol each for 10 min. After drying the substrates were subjected to UV-ozone treatment for 15 min. Subsequently, the SnO_2_ diluted solution was spin-coated at 3000 rpm for 30 s and then thermal annealed at 150 °C for 15 min. Prior to the active film coatings the substrates were subjected to the UV treatment for 10 min. A solution of the polymers with Y6 was made by dissolving in CHCl_3_ with 0.5% DIO. The blended solution was spin-coated at 2000–3000 rpm for 30 s to achieve an active layer thickness of 100 nm. Post process of spin-coating, the substrates were taken under a pressure of < 10^–6^ Torr, with 8 nm and 100 nm thickness of MoO_x_ and Ag metal electrode, respectively using thermal evaporator.

### Photovoltaic properties

**Figure 4:**
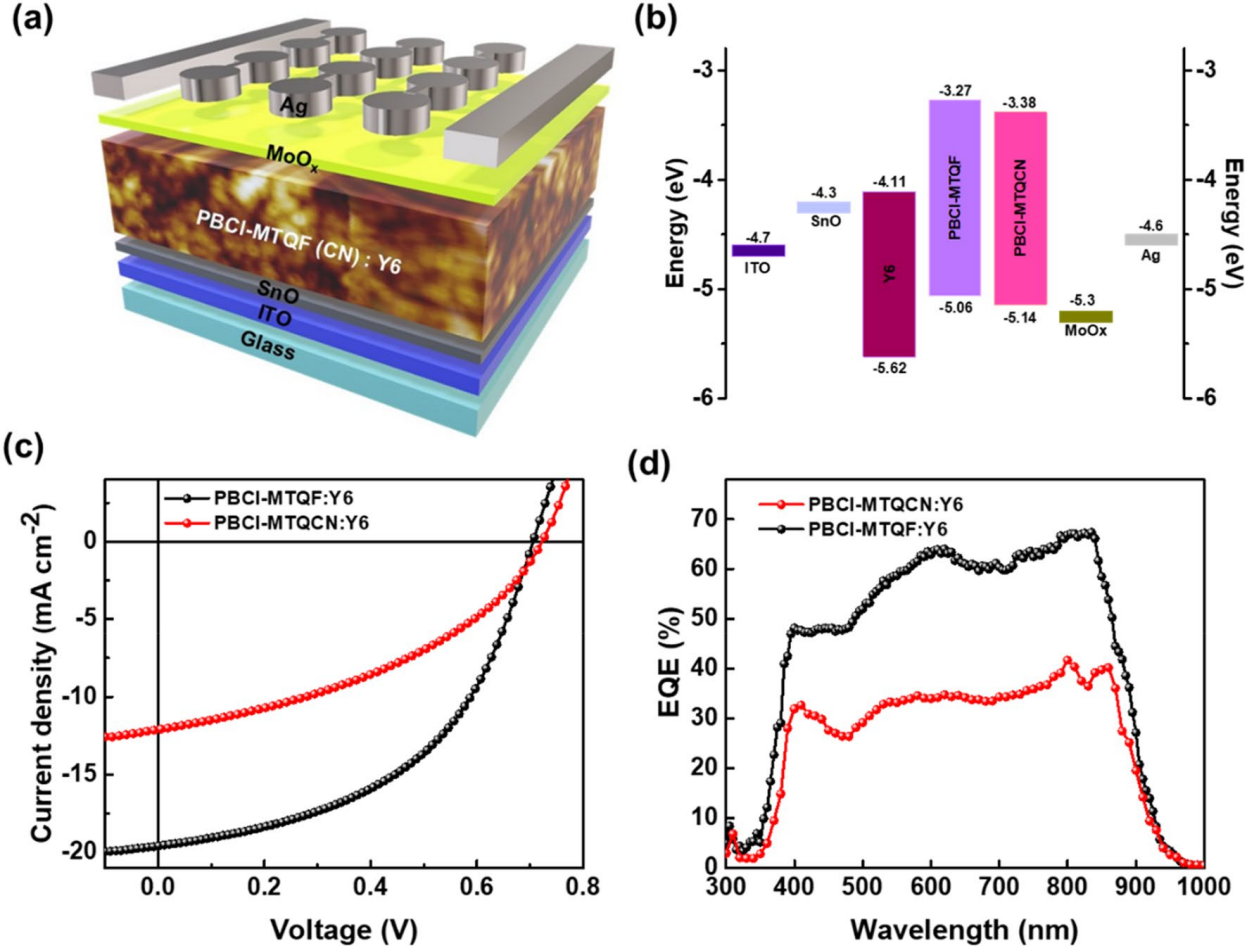
(**a**) Structure, (**b**) energy band diagram, (**c**) *J*–*V* curves and (**d**) EQE curves of inverted-type device based on PBCl-MTQF and PBCl-MTQCN as polymeric donor.

To investigate the effects of the CN and F substituents on the photovoltaic properties of the polymer, we introduced an inverted device configuration of ITO/SnO/polymer:Y6/MoO_3_/Ag (Fig. 4a). Because Y6 is one of the best non-fullerene acceptors for sufficient light absorption and efficient charge transport^5^, we selected Y6 as an electron acceptor for device fabrication. As shown in Fig. 4b, the HOMO and LUMO levels of both polymers agreed well with those of Y6 for cascade energy level alignment. For device optimization, various D:A ratios of the active layers of the devices were tested (Fig. S1 and Table S1 in the ESI). The optimum devices comprising PBCl-MTQF and PBCl-MTQCN had D:A ratios of 1:1.5, in the presence of 1,8-diiodooctane as a processing additive. The current density–voltage (*J*–*V*) characteristics and the external quantum efficiency (EQE) of the optimized devices are shown in Fig. 4c,d, respectively. The corresponding photovoltaic parameters are presented in Table 2. It was discovered that the device based on PBCl-MTQF exhibited a higher PCE of 7.48% with a short-circuit current density (*J*_*SC*_) of 19.26 mA/cm^2^, open-circuit voltage (*V*_*OC*_) of 0.71 V, and fill factor (*FF*) of 0.54. By contrast, the device based on PBCl-MTQCN indicated lower values for their photovoltaic parameters (PCE: 3.52%; *J*_*SC*_: 12.07 mA/cm^2^, *V*_*OC*_: 0.72; and *FF*: 0.40). In addition, the EQE curves of both devices encompassed broad wavelengths ranging from 350 to 950 nm (Fig. 4d). The maximum EQE value of the device based on PBCl-MTQF was 67% higher than that of the device based on PBCl-MTQCN (42%). The calculated *J*_*SC*_ values from the EQE curves were consistent with those obtained from the *J*–*V* characteristics. These results demonstrate the significant dependence of the photovoltaic performance of D-A-type quinoxaline-based polymers on the type of electron-withdrawing substituent on the quinoxaline acceptor.

**Table 2:** Best photovoltaic parameters of PSCs. Values in parentheses represent average (of 10 devices) value of photovoltaic parameters for each device.

Polymer	Blend ratio (Polymer:Y6)	*J*_SC_ (mA/cm^2^)	*J*_SC_ (mA/cm^2^)^a^	*V*_OC_ (V)	*FF*	*PCE* (%)
PBCl-MTQF	1:1.5	19.26	19.13	0.71	0.54	7.48 (7.21 ± 0.20)^b^
PBCl-MTQCN	1:1.5	12.07	11.48	0.72	0.40	3.52 (3.31 ± 0.21)^b^

^a^Calculated from EQE curves of the devices.

^b^Values in parenthesis mean average PCE.

### Charge recombination characteristics

**Figure 5:**
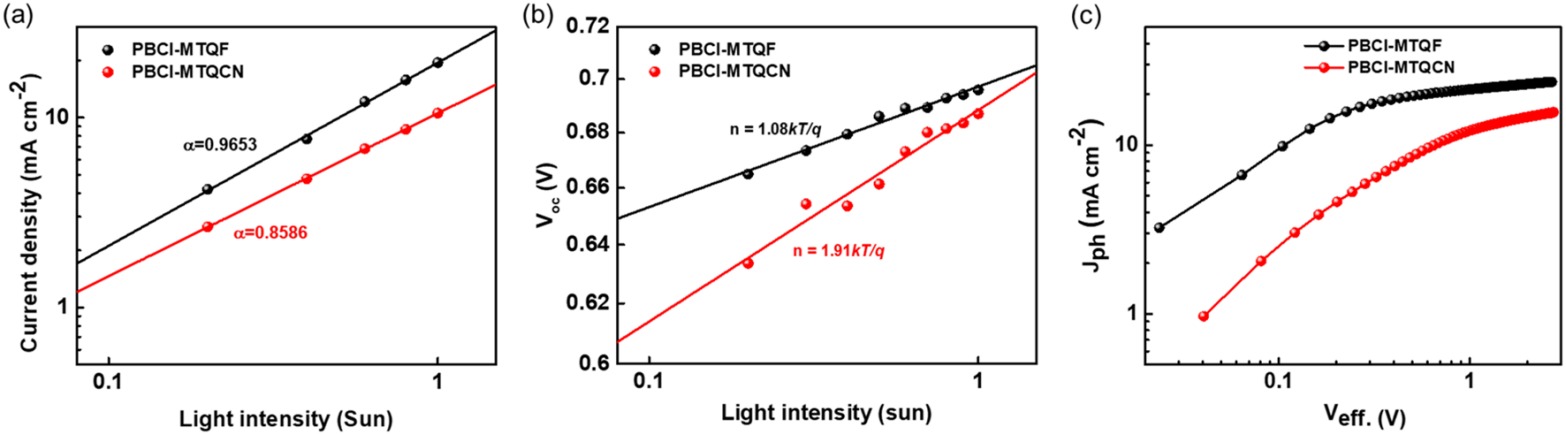
(**a**) *Jsc* vs. light intensity plots and (**b**) *Voc* vs. light intensity plots of devices. (**c**) *J*_ph_–*V*_eff_ curves of polymers.

To further evaluate the charge transport characteristics of the active blends in single-carrier devices, *J*–*V* characteristics of the electron- and hole-only devices were analyzed with a well-known space-charge-limited-current model^30^. The structures of the devices employed were ITO/SnO/active layer/LiF/Al and ITO/PEDOT:PSS/active layer/Au for the electron- and hole-only devices, respectively. The characteristic *J*–*V* curves of the single-carrier devices are shown in Fig. S2 and Table S2 in the ESI. The electron (*µ*_*e*_) and hole (*µ*_*h*_) mobilities of the PBCl-MTQF:Y6 device were measured to be 1.8 × 10^–4^ and 3.2 × 10^–4^ cm^2^ V^−1^ s^−1^, whereas those of the PBCl-MTQCN:Y6 device were 1.2 × 10^–4^ and 2.0 × 10^–4^ cm^2^ V^−1^ s^−1^, respectively. The replacement of F atom in polymer donors with the stronger electron-withdrawing CN group can often induce the detrimental effects on the charge-carrier mobilities of the devices such as the decrease in exciton splitting efficiency and increase in undesirable charge recombination process^31,32^. The decreased charge transport ability of the PBCl-MTQCN:Y6 device might have contributed to the lower *J*_*SC*_ values. Owing to the high *µ*_*e*_ and *µ*_*h*_ values of PBCl-MTQF:Y6, high values of *J*_*SC*_ and *FF* were observed, which might have promoted charge transport in the device performance. To compare the charge recombination behaviors of the devices comprising PBCl-MTQF and PBCl-MTQCN, we measured the dependence of *J*_SC_ and *V*_OC_ on light intensity. Figure 5a,b show the *J*_SC_ and *V*_OC_ dependence curves as a function of light intensity, respectively. Based on the power-law relationship, the *J*_*SC*_ vs. light intensity (*P*_*light*_) can be described as *J*_*SC ∝*_* P*_*light*_^*α*^*,* where *α* is the power-law factor and reflects the tendency of bimolecular recombination^33^. The *α* value of approximately unity in the log–log plot of *J*_*SC*_ vs. light intensity indicates weak bimolecular recombination under short-circuit conditions^34^. As shown in Fig. 5a, the PBCl-MTQF and PBCl-MTQCN devices exhibited α values of 0.97 and 0.86, respectively. These results indicate the suppression of undesirable bimolecular recombination loss in the PBCl-MTQF device, whereas dominant bimolecular charge-carrier recombination loss was observed in the PBCl-MTQCN device during charge transport.

The relationship between the *V*_OC_ of the devices and light intensity can be expressed as *V*_oc_ = (*n*kT/q) × ln[$\frac{{J}_{ph}}{{J}_{o}}+1$], where *n* is the ideality factor, *k* the Boltzmann constant, *T* the absolute temperature, and *q* the elementary charge. The slope from the *V*_OC_–light intensity curve provides information regarding the trap-assisted recombination loss within the device^35,36^. As shown in Fig. 5b, the devices comprising PBCl-MTQF and PBCl-MTQCN exhibited *n* values of 1.08 *kT/q* and 1.91 *kT/*q, respectively. The PBCl-MTQF device exhibited a lower *n* value, indicating reduced trap-assisted recombination during charge transport. By contrast, the introduction of CN groups can significantly increase the population of trap-assisted recombination, thereby decreasing the charge mobility in the device. Weak bimolecular and trap-assisted recombination losses enable higher efficiencies in devices based on PBCl-MTQF.

To further evaluate the exciton dissociation and charge extraction properties, we analyzed the correlation between the photocurrent density (*J*_*ph*_) and effective voltage (*V*_*eff*_), where *J*_*ph*_ = *J*_*L*_ (current density under illumination) − *J*_*D*_ (current density in dark) and *V*_*eff*_ = *V*_*0*_ (voltage at *J*_*ph*_ = 0) − *V*_*a*_ (applied voltage) (Fig. 5c). At V_eff_ ≫ 2.0 V, the saturated current densities (*J*_*sat*_) values of devices PBCl-MTQF and PBCl-MTQCN with Y6 were calculated to be 23.67 and 15.67 mA cm^−2^, respectively (Fig. 5c). Under the short-circuit conditions, the dissociation probability [*P(E,T)*] at the D/A interfaces was calculated to be 86.27% (PBCl-MTQF) and 67.81% (PBCl-MTQCN)^37^. Additionally, the maximum charge collection efficiency of the devices with PBCl-MTQF and PBCl-MTQCN were 67.81% and 41.25%, respectively. The high *J*_*sc*_ and PCE values of the PBCl-MTQF device was due to the efficient exciton dissociation and charge collection efficiency. In general, the device comprising PBCl-MTQF exhibited better charge recombination and charge extraction properties compared with the device comprising PBCl-MTQCN.

### Morphology of blended films

**Figure 6:**
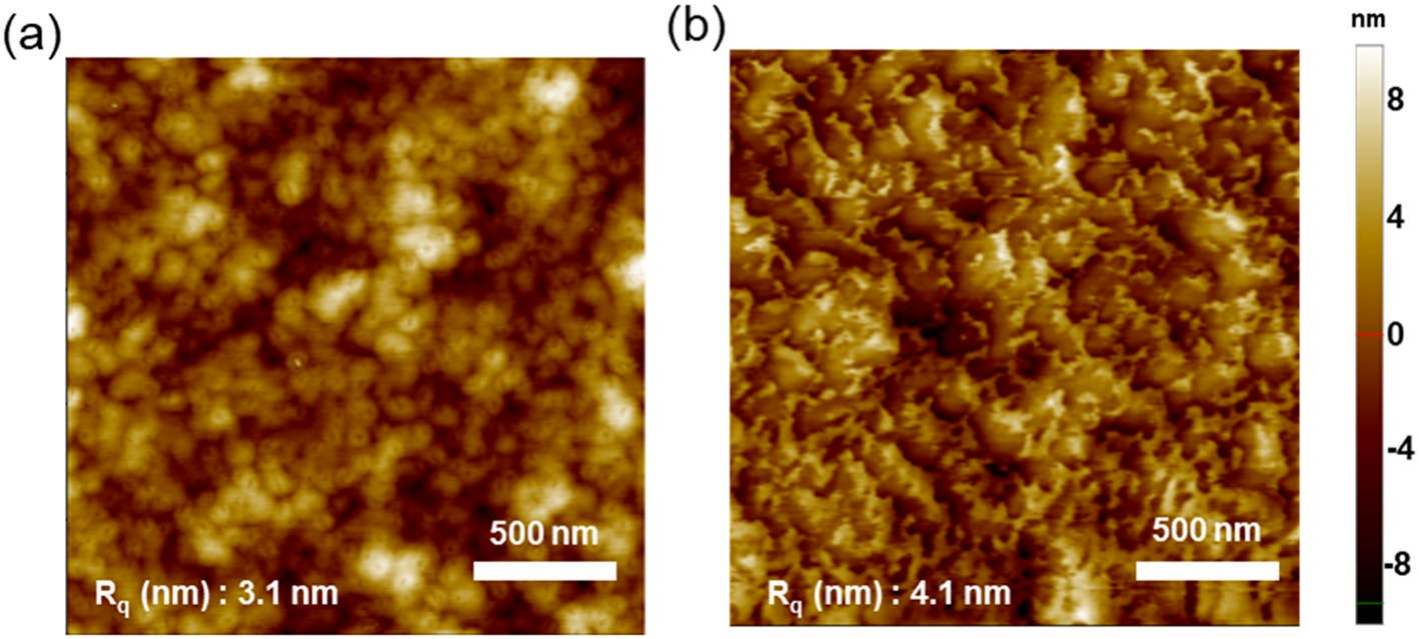
Tapping-mode AFM images of blend films of (**a**) PBCl-MTQF and (**b**) PBCl-MTQCN with Y6.

To analyze the surface morphology of the active layer, atomic force microscopy (AFM) measurements were performed in the tapping mode, and the resultant images are shown in Fig. 6. The polymer:Y6 blend films based on different polymers showed distinct morphological differences. A homogeneous spherical morphology was observed for the blend film comprising PBCl-MTQF, whereas the film comprising PBCl-MTQCN exhibited an irregular granular morphology. Consequently, the blend film comprising PBCl-MTQF exhibited a relatively uniform film and contained bicontinuous interpenetrating networks, as compared with the film comprising PBCl-MTQCN. The larger domain size of the film comprising PBCl-MTQCN yielded a high root-mean-square (RMS) surface roughness of 4.1 nm, compared with that of the film comprising PBCl-MTQF (RMS value of 3.1 nm). The introduction of CN-groups in the polymer chain resulted in the formation of larger DA domains, which hindered efficient exciton dissociation and charge transport.

**Figure 7:**
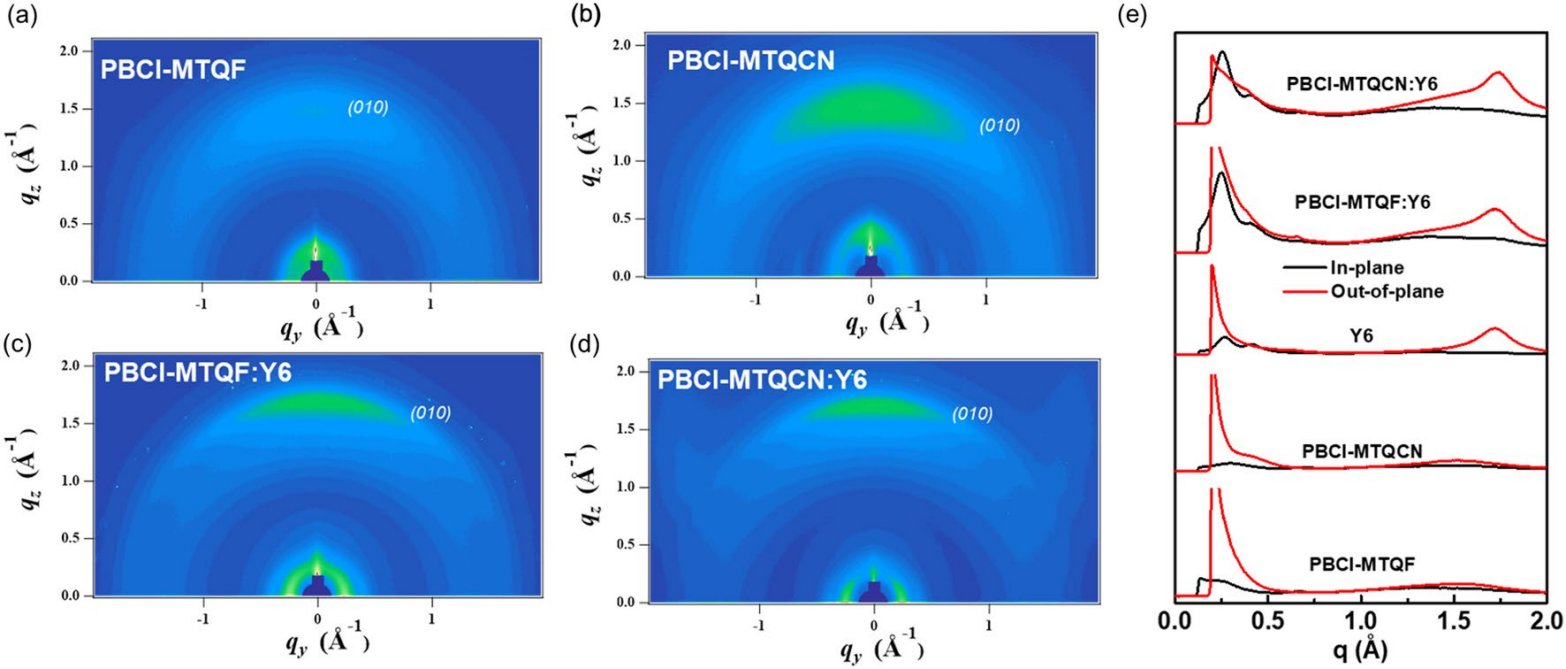
GIWAXS patterns of (**a**,**b**) neat films and (**c**,**d**) blend films of PBCl-MTQF and PBCl-MTQCN. (**e**) Line-cut profiles of neat and blend films (red line: out-of-plane; black line: in-plane).

To investigate the molecular orientation and crystallinity of the neat and blended films, grazing incidence X-ray scattering (GIWAXS) measurements were performed. Their GIWAXS patterns and corresponding line-cut profiles are shown in Fig. 7. The corresponding GIWAXS parameters are listed in Table S3 in the ESI. The neat PBCl-MTQF film exhibited an extremely weak π–π stacking (010) peak at 1.54 Å^−1^ in the out-of-plane (OOP) direction and multiple lamellar packing (h00) diffraction peaks in the in-plane (IP) direction (Fig. 7a). This suggests that the PBCl-MTQF film exhibited different packing modes with moderate crystalline features, indicating a moderate face-on orientation in favor of charge transport. By contrast, the neat PBCl-MTQCN film exhibited a relatively clear π–π stacking (010) peak at 1.51 Å^−1^ and multiple lamellar packing (h00) peaks in the OOP and IP directions, respectively (Fig. 7b), indicating the stronger crystalline properties of PBCl-MTQCN compared with that of PBCl-MTQF in the pristine polymer state. Moreover, pristine PBCl-MTQF and PBCl-MTQCN films showed different crystal coherence length (CCL) of 6.25 and 7.67 Å, respectively, suggesting the stronger crystallinity of PBCl-MTQCN. The neat film of Y6 exhibited a strong π–π stacking diffraction peak in the OOP direction at 1.72 Å^−1^ (d = 3.65 Å^−1^), and two other diffraction peaks at q = 0.27 and 0.415 Å^−1^ in the IP direction (Fig. S3 in ESI). This suggests the coexistence of two different ordered structures in Y6.

The GIWAXS analysis of the blended films revealed that both blend films exhibited a lamellar diffraction peak (100) along the IP direction and a clear π–π stacking diffraction peak (010) in the OOP direction (Fig. 7c,d). The GIWAXS pattern of the PBCl-MTQF:Y6 blend film showed a dominant diffraction peak for Y6 (Fig. S3 in ESI). These results agreed relatively well with those of the pristine PBCl-MTQF film as compared with those of PBCl-MTQCN. Hence, a more homogeneous mixing between PBCl-MTQF and Y6 can be expected in the blend film, which is consistent with the AFM results (Fig. 6a). The CCL values of the pristine films confirmed the strong internal π–π stacking in PBCl-MTQCN compared with that of PBCl-MTQF, which indicates a stronger intramolecular interaction in PBCl-MTQCN. By contrast, the blends of PBCl-MTQF:Y6 showed an enhanced crystalline phase with coherence to the pristine Y6 diffraction peaks; this yielded a more preferable face-on molecular orientation packing, which is advantageous for charge transport.

## Conclusion

Two-dimensional quinoxaline-based D–A type polymers of PBCl-MTQF and PBCl-MTQCN, in which the strong electron-withdrawing substituents of F and CN units were selectively incorporated, respectively, were synthesized via the Stille coupling reaction to investigate their effects on the photovoltaic properties of the polymers. Owing to the higher electron-withdrawing capability of the CN moiety compared with that of the F atom, PBCl-MTQCN exhibited not only a higher *ε* value in the ICT region, but also a lower HOMO energy level compared with PBCl-MTQF. Under these conditions, the concomitant enhancement in the *Jsc*, *Voc*, and PCE of the PBCl-MTQCN-based device can be anticipated. However, a higher PCE of 7.48% was achieved from the PBCl-MTQF-based device, which exhibited a *Jsc* of 19.26 mA/cm^2^, *Voc* of 0.71 V, and FF of 0.54. The PCE of the PBCl-MTQCN-based device was restricted to 3.52%, with a *Jsc* of 12.07 mA/cm^2^, *Voc* of 0.72 V, and FF of 0.40. Hence, it was clear that the *Jsc*, *Voc*, and PCE of the PBCl-MTQCN-based device were inferior to those of the PBCl-MTQF-based device. The significant reduction in the *Jsc* and FF of the PBCl-MTQCN-based device was attributable to the unfavorable aggregation-induced charge recombination and charge transport kinetics. Owing to the high aggregation behavior of PBCl-MTQCN, as revealed via AFM, a significant amount of undesirable bimolecular recombination loss was observed during charge transport. Therefore, the incorporation of electron-withdrawing substituents on the quinoxaline-based polymers must be controlled well because their photovoltaic properties are affected significantly by the type of existing substituents. This study provides valuable information regarding the structure–property relationships of D–A-type quinoxaline-based polymers for photovoltaic applications.

## Supplementary Information

Supplementary Information.
