# Supplementary material for: Effect of electron-withdrawing fluorine and cyano substituents on photovoltaic properties of two-dimensional quinoxaline-based polymers
Source: Sci Rep. 2021 Dec 21;11:24381. doi: 10.1038/s41598-021-03763-1 (PMC8692587; doi:10.1038/s41598-021-03763-1)
Supplement: Supplementary file 1 — Supplementary Information. [file 41598_2021_3763_MOESM1_ESM.docx]

**Electronic Supporting Information (ESI) for:**

**Effect of Electron-Withdrawing Fluorine and Cyano Substituents on Photovoltaic Properties of Two-Dimensional Quinoxaline-Based Polymers**

Seok Woo Lee,^a†^ MD. Waseem Hussain,^b†^ Sanchari Shome,^b^ Su Ryong Ha,^b^ Jae Taek Oh,^b^ Dong Ryeol Whang,^c^ Yunseul Kim, ^d^ Dong-Yu Kim,^d^ Hyosung Choi,*^b^ and Dong Wook Chang*^a^

***^a^****Department of Industrial Chemistry, Pukyong National University, 48513 Busan, Republic of Korea*

*E-mail: dwchang@pknu.ac.kr*

*^b^Department of Chemistry, Research Institute for Natural Science and Institute of Nano Science and Technology, Hanyang University, 04730 Seoul, Republic of Korea*

*E-mail: hschoi202@hanyang.ac.kr*

*^c^Department of Advanced Materials, Hannam University, Daejeon, 34054 Republic of Korea.*

*^d^School of Materials Science and Engineering (SMSE), Research Institute of Solar and Sustainable Energies (RISE), Gwangju Institute of Science and Technology (GIST), Gwangju 61005, Republic of Korea*

^†^S. W. Lee and MD. W. Hussain equally contributed to this research

# Experimental methods

# Materials and Instruments

Pd(PPh_3_)_2_Cl_2,_ oxalyl chloride, and aluminium chloride were purchased from TCI chemicals. All other reagent and solvents were purchased from Aldrich Chemical Co., Inc. 4,7-bis(5-bromothiophen-2-yl)-5-fluorobenzo[c][1,2,5]thiadiazole (3), 4,7-bis(5-bromothiophen-2-yl)benzo[c][1,2,5]thiadiazole-5-carbonitrile (4), and (4,8-bis(5-(2-ethylhexyl)-4-chlorothiophen-2-yl)benzo-[1,2-b:4,5-b']dithiophene-2,6-diyl)bis(trimethylstannane) (7) were produced according to previously reported method [S1–S3]. ^1^H and ^13^C nuclear magnetic resonance (NMR) spectra were measured with a JEOL JNM ECP-400 spectrometer. UV-visible spectra were recorded on a Lamda 365 spectrophotometer. Matrix-assisted laser desorption/ionization time-of-flight (MALDI-TOF) spectroscopy was conducted by using a Bruker Ultraflex spectrometer. Gel Permeation Chromatography was analysed in o-dichlorobenzene solvent by using Shimadzu CTO-20A series instrument. Cyclic voltammetry (CV) measurements were carried out by using a VersaSTAT3 potentiostat (Princeton Applied Research) with tetrabutylammonium hexafluorophosphate (0.1M, Bu_4_NPF_6_) as the electrolyte in acetonitrile. For CV measurements, a glassy carbon electrode coated with the polymer and a platinum wire were used as the working and counter electrode, respectively. A silver wire was used as a pseudo-reference electrode with a ferrocene(Fc)/ferrocenium(Fc^+^) external standard.

**Synthesis of materials**

*4-methoxy-N-(4-methoxyphenyl)-N-phenylaniline (****1****)*

In a round bottom flask, 4-iodoanisole (17 mmol, 2.5 equiv.), aniline (6.8 mmol, 1 equiv.), and 1,10-phenanthroline (1.36 mmol, 0.2 equiv.) were dissolved in 25 mL toluene. The solution was heated to 100 ºC under nitrogen protection, and CuI (1.36 mmol, 0.2 equiv.) and KOH (68 mmol, 10 equiv.) were added rapidly. After further stirring overnight, the reaction was completed. The mixture was poured into water and the organic product was extracted with dichloromethane. After drying over magnesium sulfate, the solution was separated by filtration and the solvent was removed under reduced pressure. The crude residue was purified by column chromatography using dichloromethane/hexane (1/2, v/v) as eluent. Yield = 55% (pale yellow solid). ^1^H-NMR (400 MHz, CDCl_3_) δ 7.15 (dd, J = 8.7, 7.3 Hz, 2H), 7.03-7.00 (m, 4H), 6.91 (dd, J = 8.9, 1.1 Hz, 2H), 6.86-6.78 (m, 5H), 3.77 (s, 6H).

*1,2-bis(4-(bis(4-methoxyphenyl)amino)phenyl)ethane-1,2-dione (****2****)*

In a round bottom flask, AlCl_3_ (2.28 mmol, 2.1 equiv.) was dissolved in 20 mL anhydrous dichloroethane under nitrogen protection. After cooling down to 0 ºC, oxalyl chloride (1.10 mmol, 1 equiv.) and **1** (3.27 mmol, 3 equiv.) was added quickly. The mixture was refluxed for overnight. After completion of the reaction, the mixture was was poured into water and the organic product was extracted with dichloromethane. After drying over magnesium sulfate, the solution was separated by filtration and the solvent was removed under reduced pressure. The crude residue was purified by column chromatography using etheyl acetate/hexane (1/4, v/v) as eluent. Yield = 80% (yellow solid). ^1^H-NMR (400 MHz, CDCl_3_) δ 7.70 (d, J = 9.1 Hz, 0H), 7.09 (dd, J = 6.9, 2.3 Hz, 1H), 6.85 (dd, J = 6.9, 2.3 Hz, 1H), 6.76 (d, J = 8.7 Hz, 0H), 3.79 (s, 1H). ^13^C-NMR (100 MHz, CDCl_3_) δ 157.4, 154.1, 138.8, 131.8, 128.1, 124.2, 116.7, 115.1, 55.6

*4,4'-(5,8-bis(5-bromothiophen-2-yl)-6-fluoroquinoxaline-2,3-diyl)bis(N,N-bis(4-methoxyphenyl)aniline) (****5****)*

A mixture of 4,7-bis(5-bromothiophen-2-yl)-5-fluorobenzo[c][1,2,5]thiadiazole (**3**, 0.361 mmol, 1.2 equiv.) and zinc powder (7.22 mmol, 20 equiv.) in acetic acid (20 mL) was stirred at 80 ºC for 2 h. Upon completion of the reaction, zinc power was removed by filtration and the filtrate was collected. After addition of **2** (0.30 mmol, 1 equiv.) to the filtrate, the mixture was heated to reflux overnight. The solution was cooled to room temperature, and the mixture was poured into water and extracted with chloroform. The organic phase was separated, dried over magnesium sulfate, and filtered. Solvents were removed under reduced pressure, and the crude residue was purified by column chromatography using methylene chloride/hexane (2/1, v/v) as eluent. Yield = 62% (yellow solid). ^1^H-NMR (400 MHz, CDCl_3_) δ 7.79 (d, J = 13.7 Hz, 1H), 7.71 (q, J = 1.8 Hz, 1H), 7.58 (td, J = 6.6, 2.0 Hz, 4H), 7.50 (d, J = 4.1 Hz, 1H), 7.15-7.09 (m, 10H), 6.90-6.83 (m, 12H), 3.80 (s, 12H). ^13^C-NMR (100 MHz, CDCl_3_) δ 157.3, 156.5, 156.5, 152.4, 151.1, 149.9, 149.7, 140.2, 140.2, 138.7, 137.7, 137.6, 133.8, 133.5, 131.4, 131.2, 131.0, 130.9, 130.3, 130.1, 129.4, 129.2, 127.5, 127.5, 126.1, 118.5, 118.4, 118.0, 117.4, 115.2, 114.9, 55.6. MALDI-TOF MS: m/z calcd, 1076.09; found, 1077.065 [M^+^].

2,3-bis(4-(bis(4-methoxyphenyl)amino)phenyl)-5,8-bis(5-bromothiophen-2-yl)quinoxaline-6-carbonitrile (**6**)

The same procedure used to prepare **5** was used to produce **6**. Instead of **3**, 4,7-bis(5-bromothiophen-2-yl)benzo[c][1,2,5]thiadiazole-5-carbonitrile (**4**) was used as the starting material. Meanwhile, the crude product was purified by column chromatography using dichloromethane/hexane (4/1, v/v) as eluent. Yield = 50% (red solid). ^1^H-NMR (400 MHz, CDCl_3_) δ 8.14 (s, 1H), 7.81 (d, J = 4.1 Hz, 1H), 7.64 (d, J = 9.1 Hz, 2H), 7.58 (d, J = 8.7 Hz, 2H), 7.53 (d, J = 4.1 Hz, 1H), 7.18-7.12 (m, 10H), 6.89-6.84 (m, 12H), 3.81 (d, J = 1.8 Hz, 12H). ^13^C-NMR (100 MHz, CDCl_3_) δ 156.8, 156.7, 153.2, 150.5, 150.3, 139.9, 139.8, 138.1, 136.8, 135.8, 134.2, 131.5, 131.3, 131.2, 130.5, 129.5, 128.7, 128.5, 128.0, 127.7, 127.6, 126.5, 118.3, 118.1, 118.0, 114.9, 114.9, 109.0, 55.6. MALDI-TOF MS: m/z calcd, 1083.09; found, 1084.017 [M^+^].

***PBCl-MTQF***

In a Schlenk flask, **5** (0.14 mmol, 1 equiv.), (4,8-bis(4-chloro-5-(2-ethylhexyl)thiophen-2-yl)benzo[1,2-b:4,5-b']dithiophene-2,6-diyl)bis(trimethylstannane) (**7**, 0.14 mmol, 1 equiv.), tri(*o*-tolyl)phosphine (0.056 mmol, 40 mol%) and tris(dibenzylideneacetone)dipalladium (Pd_2_(dba)_3_, 0.007 mmol, 5 mol%) were mixed together in anhydrous mixed solvent of chlorobenzene (4 mL) and DMF (1 mL). After nitrogen bubbling for 15 min, the solution was stirred at 110 ºC for 48 h under nitrogen protection. The polymerization was finished by adding two end-capping agents of 2-trimethylstannylthiophene and 2-bromothiophene at 2h interval. The polymer solution was precipitated in methanol and the solid was collected by filtration. The solid residue was further purified by Soxhlet extraction with methanol, acetone, hexane, and chloroform. The polymer in chloroform faction was recovered by precipitation into methanol again, Finally, the polymer was dried in a vacuum oven at 50 ºC. Yield = 92% (dark purple solid). ^1^H-NMR (400 MHz, CDCl_3_) δ 7.54-7.85 (5H), 7.30-7.52 (2H), 6.98-7.19 (12H), 6.72-6.93 (14H), 3.62-3.82 (12H), 2.65-3.00 (4H), 1.69-1.83 (2H), 1.23-1.45 (16H), 0.84-0.99 (12H). Molecular weight by GPC: number-average molecular weight (Mn) = 20.82 KDa, polydispersity index (PDI) = 2.48.

***PBCl-MTQCN***

The same procedure used to prepare **PBCl-MTQF** was used to produce **PBCl-MTQCl**. Instead of **5**, **6** was used as the alternative dibrominated quinoxaline monomer. Yield = 96% (dark purple solid). ^1^H-NMR (400 MHz, CDCl_3_) δ 7.83-8.05 (2H), 7.36-7.76 (5H), 6.91-7.22 (12H), 6.58-6.91 (12H), 6.21-6.41 (2H), 3.48-3.95 (12H), 3.16-3.33 (2H), 2.76-2.96 (2H), 1.68-1.81 (2H), 1.20-1.48 (16H), 0.67-1.03 (12H) Molecular weight by GPC: number-average molecular weight (Mn) = 26.23 KDa, polydispersity index (PDI) = 2.24.

**
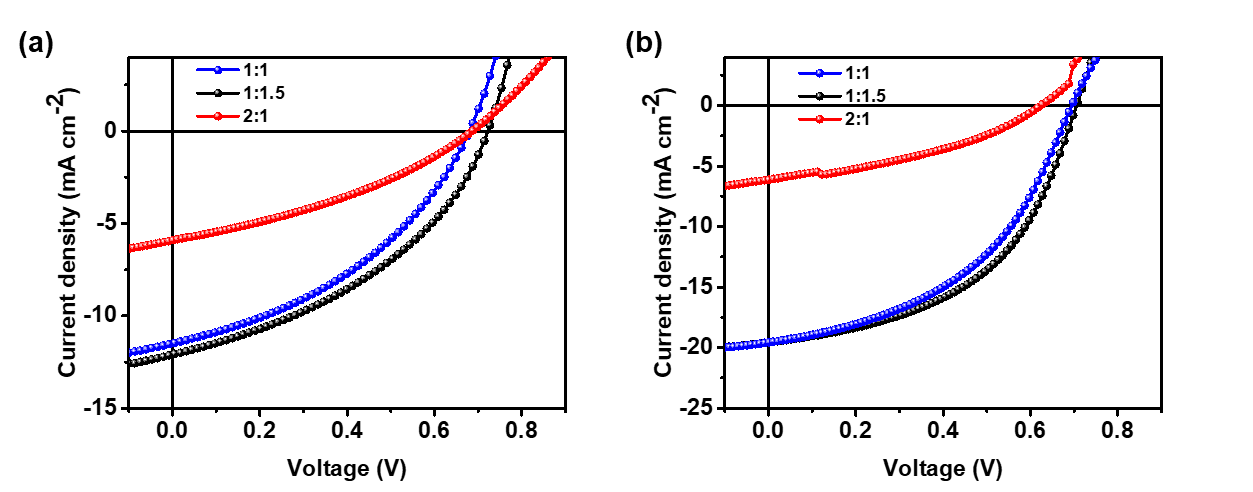
**

**Figure S1.** *J-V* curves of the devices with (a) PBCl-MTQCN and (b) PBCl-MTQF as a function of D:A ratio.

**Table S1**. The detailed photovoltaic parameters of the devices with different D:A ratio.

| Polymer | D:A  ratio | Spin rate   (rpm) | Jsc  (mA cm^-2^) | Voc  (V) | FF | PCE  (%) |
| --- | --- | --- | --- | --- | --- | --- |
| PBCl-MTQF | 1:1.5 | 3500 | 19.26 | 0.71 | 0.54 | 7.48 |
|  | 1:1 | 3000 | 19.60 | 0.69 | 0.50 | 6.4 |
|  | 2:1 | 2000 | 6.10 | 0.62 | 0.38 | 1.44 |
| PBCl-MTQCN | 1:1.5 | 3000 | 12.07 | 0.72 | 0.40 | 3.52 |
|  | 1:1 | 3000 | 11.46 | 0.67 | 0.39 | 3.10 |
|  | 2:1 | 2000 | 5.90 | 0.68 | 0.34 | 1.4 |

* All devices have 1,8-diiodooctane (DIO) as an additive.

**Electron- and hole-only devices**

To investigate the electron and hole mobilities we fabricated the electron- and hole-only devices of the active films (PBCl-MTQF:Y6 and PBCl-MTQCN:Y6) with a structure ITO/SnO/active layer/LiF/Al and ITO/PEDOT:PSS/active layer/Au, respectively. The electron and hole mobilities of the device were calculated by employing the Mott-Gurney equation given below as [S4]

$J= \frac{8}{9}$µ ${}_{0}$ε$\frac{V^{2}}{d^{3}}$

where, μ, ε and ε_0_ are the charge mobility, the relative permittivity and the permittivity of free space, respectively and d represents the thickness of the layers between the electrodes. And *V = V_appl_ – V_bi_ – V_s_*, where *V_appl_*, *V_s_* and *V_bi_* refers to applied voltage, series resistance voltage and built-in voltage, respectively.

**
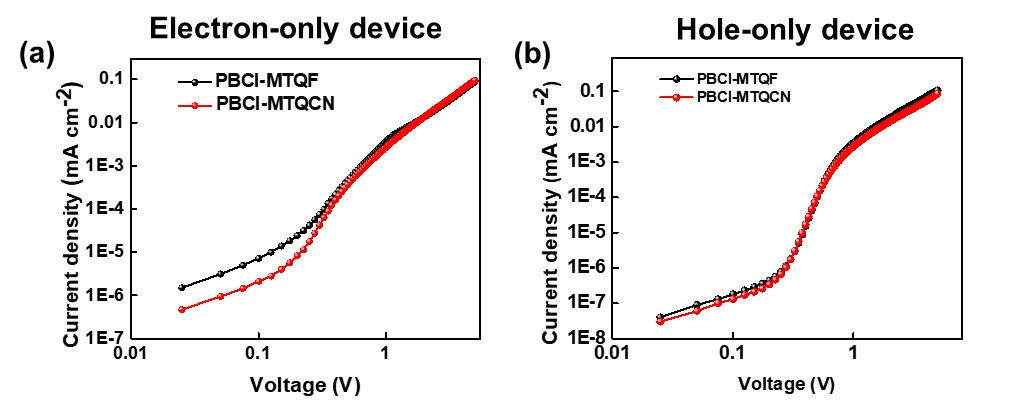
**

**Figure S2.** The current density vs voltage plots of (a) electron-only devices with structure ITO/SnO/active layer/LiF/Al and (b) hole-only devices with structure ITO/PEDOT:PSS/active layer/Au.

**Table S2.** The electron and hole mobilities calculated from the J-V characteristics of the electron- and hole-only devices.

| Active layer | Electron mobility^a^  (cm^2^/Vs) | Hole mobility^b^  (cm^2^/Vs) |
| --- | --- | --- |
| PBCl-MTQF:Y6 | 1.8 × 10^-4^ | 3.2 × 10^-4^ |
|  |  |  |
| PBCl-MTQCN:Y6 | 1.2 × 10^-4^ | 2.0 × 10^-4^ |

^a^ Electron-only device: ITO/SnO/polymer:Y6/LiF/Al

^b^ Hole-only device: ITO/PEDOT:PSS/polymer:Y6/Au

**Table S3**. Molecular packing and their characteristic length scales for the neat and blended films.


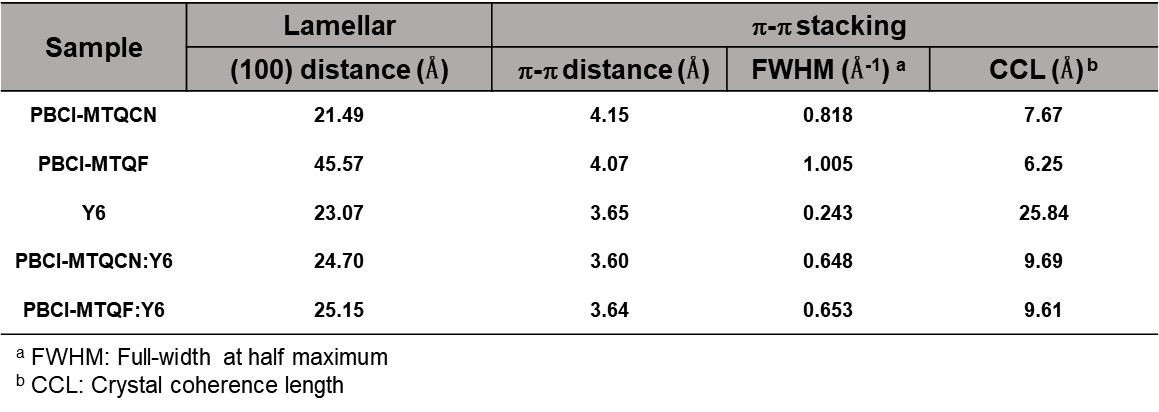


**
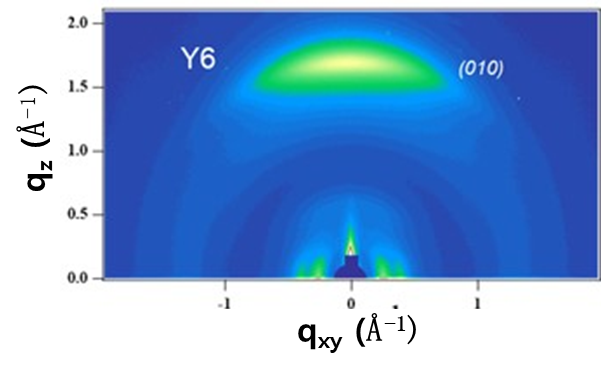
**

**Figure S3**. The 2D GIWAXS pattern for the neat Y6 film.

**[Appendix]**


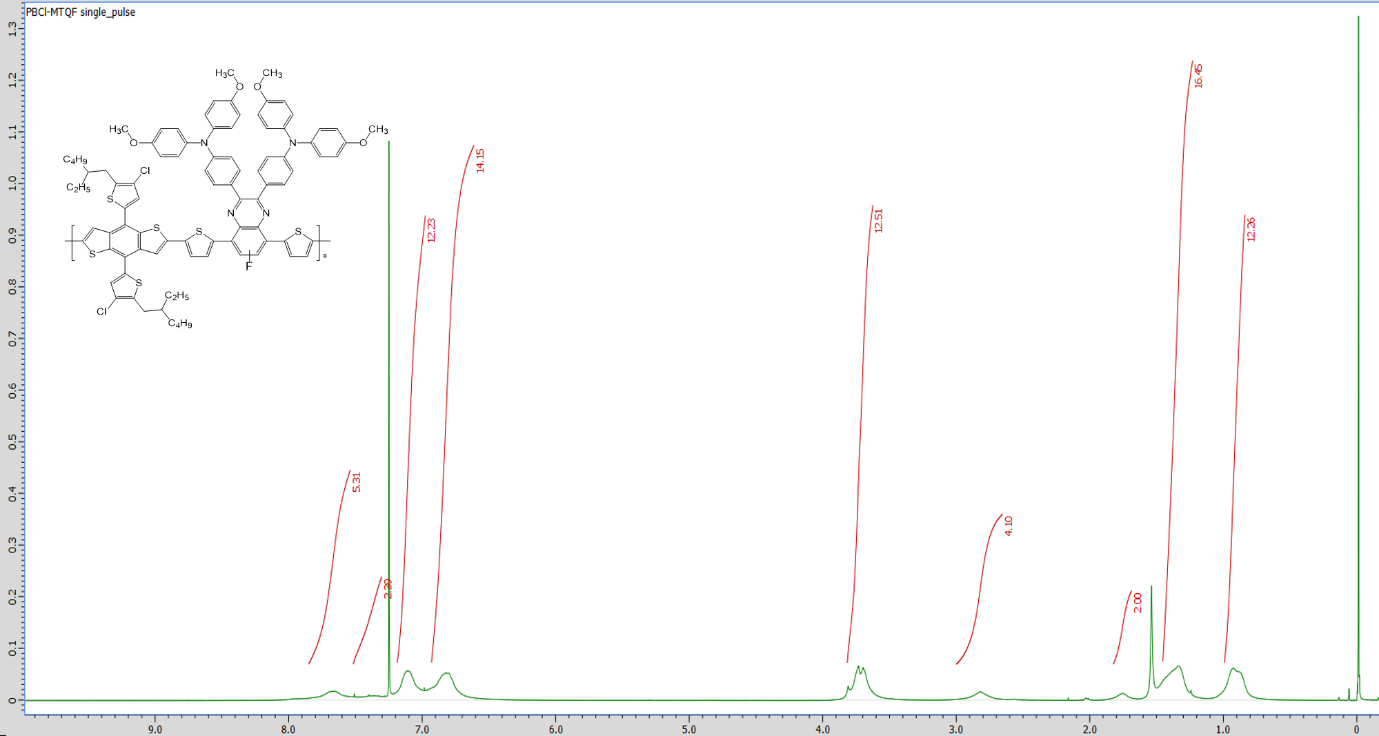


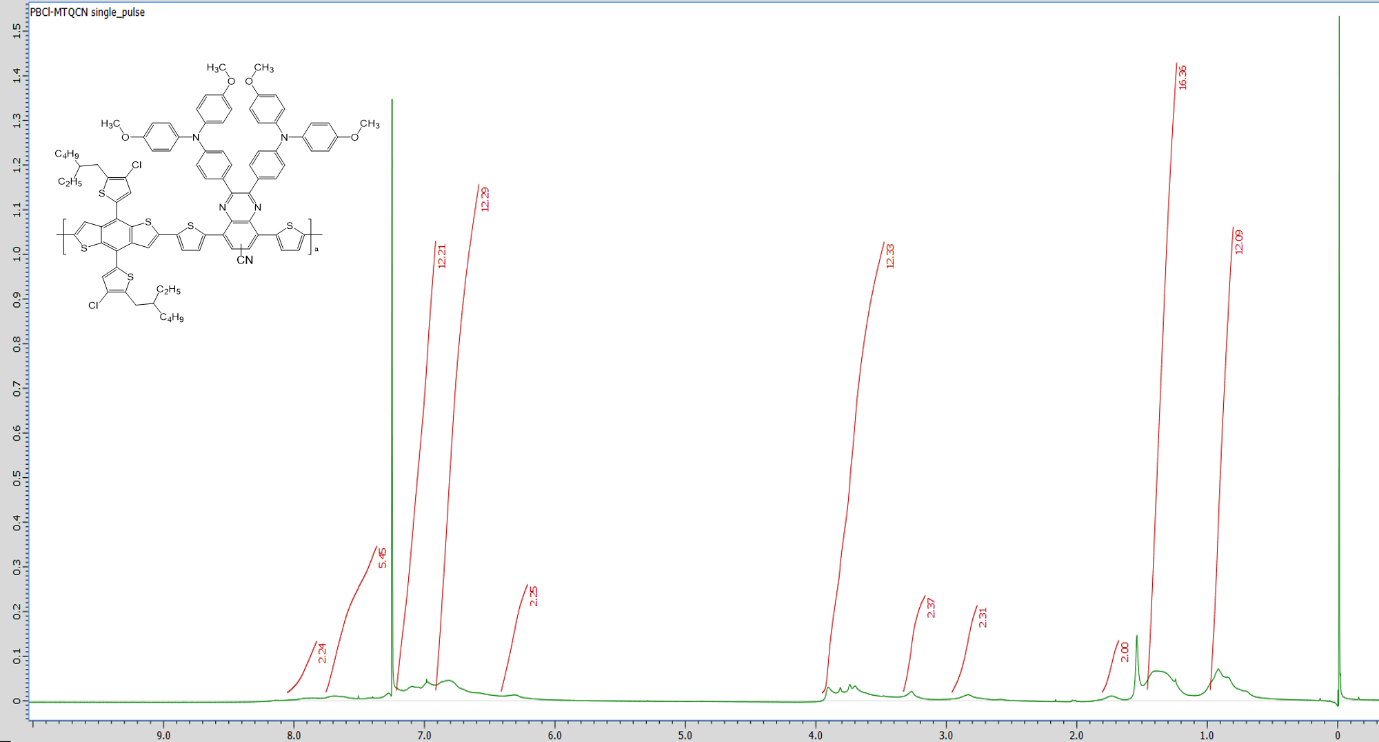


^1^H NMR Spectra of two polymers.

**
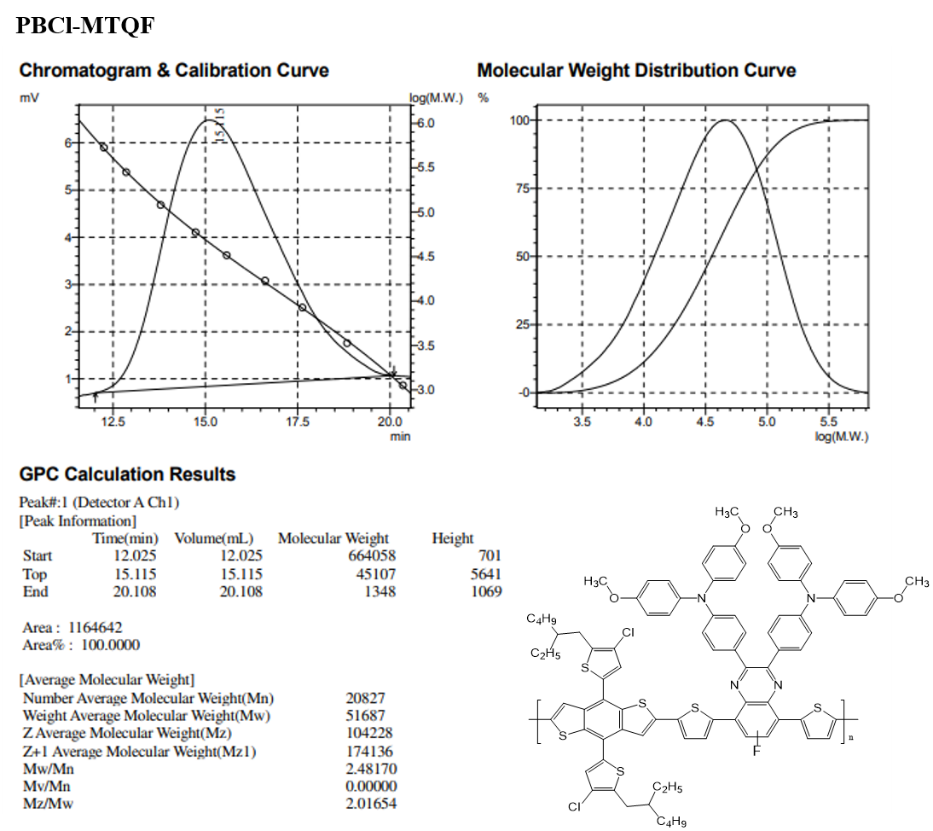
**


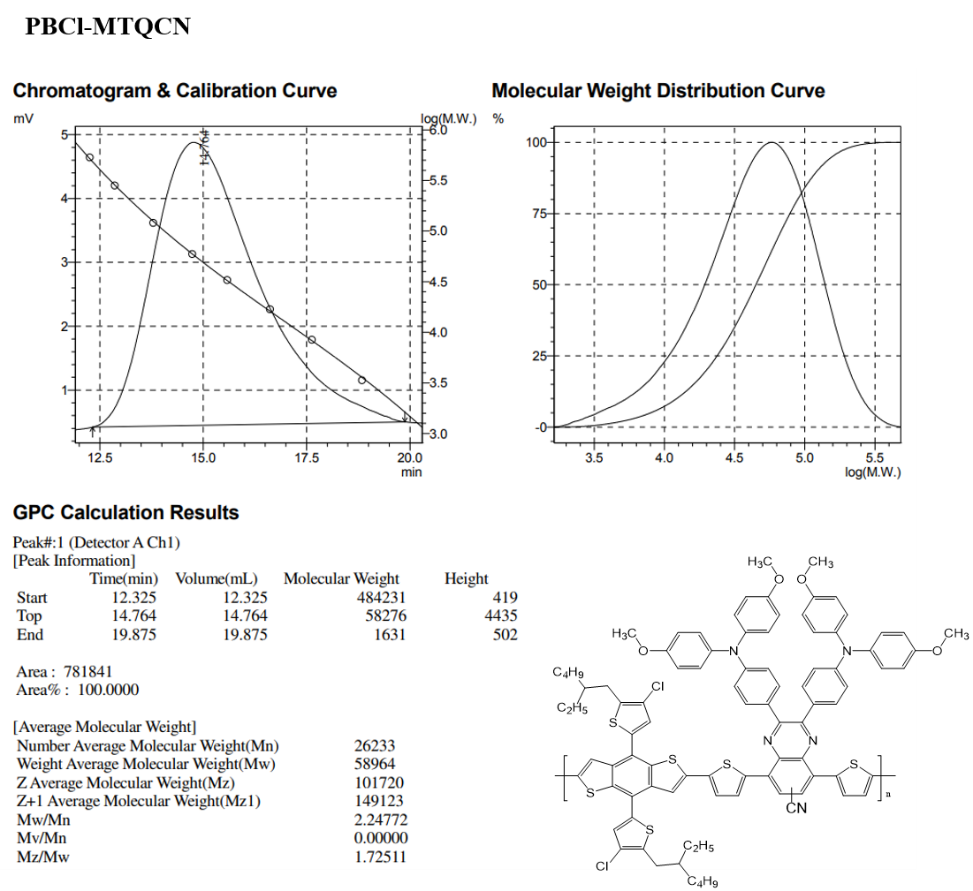


GPC analysis of two polymers.

**Reference**

S1. Kim, J. *et al.* Synthesis of PCDTBT-based fluorinated polymers for high open-circuit voltage in organic photovoltaics: Towards an understanding of relationships between polymer energy levels engineering and ideal morphology control. *ACS Applied Materials and Interfaces* **6**, 7523–7534 (2014).

S2. Zhang, S., Qin, Y., Zhu, J. & Hou, J. Over 14% Efficiency in Polymer Solar Cells Enabled by a Chlorinated Polymer Donor. *Advanced Materials* **30**, 1–7 (2018).

S3. Handoko, S. L., Jin, H. C., Whang, D. R., Kim, J. H. & Chang, D. W. Effect of cyano substituent on photovoltaic properties of quinoxaline-based polymers. *Journal of Industrial and Engineering Chemistry* **86**, 244–250 (2020).

S4. Nikolka, M. *et al.* High-mobility, trap-free charge transport in conjugated polymer diodes. *Nature Communications* **10**, 1–9 (2019).
